# Protective Effects of Nanoparticle-Loaded Aliskiren on Cardiovascular System in Spontaneously Hypertensive Rats

**DOI:** 10.3390/molecules24152710

**Published:** 2019-07-25

**Authors:** Olga Pechanova, Andrej Barta, Martina Koneracka, Vlasta Zavisova, Martina Kubovcikova, Jana Klimentova, Jozef Tӧrӧk, Anna Zemancikova, Martina Cebova

**Affiliations:** 1 Institute of Normal and Pathological Physiology, Centre of Experimental Medicine, Slovak Academy of Sciences, Sienkiewiczova 1, 813 71 Bratislava, Slovakia; 2 Institute of Experimental Physics, Slovak Academy of Sciences, Watsonova 47, 040 01 Kosice, Slovakia

**Keywords:** hypertension, renin, aliskiren, nitric oxide, heart, aorta, vasoactivity, collagen, PLA nanoparticles

## Abstract

Aliskiren, a renin inhibitor, has been shown to have cardioprotective and blood pressure (BP) lowering effects. We aimed to determine the effects of nanoparticle-loaded aliskiren on BP, nitric oxide synthase activity (NOS) and structural alterations of the heart and aorta developed due to spontaneous hypertension in rats. Twelve week-old male spontaneously hypertensive rats (SHR) were divided into the untreated group, group treated with powdered or nanoparticle-loaded aliskiren (25 mg/kg/day) and group treated with nanoparticles only for 3 weeks by gavage. BP was measured by tail-cuff plethysmography. NOS activity, eNOS and nNOS protein expressions, and collagen content were determined in both the heart and aorta. Vasoactivity of the mesenteric artery and wall thickness, inner diameter, and cross-sectional area (CSA) of the aorta were analyzed. After 3 weeks, BP was lower in both powdered and nanoparticle-loaded aliskiren groups with a more pronounced effect in the latter case. Only nanoparticle-loaded aliskiren increased the expression of nNOS along with increased NOS activity in the heart (by 30%). Moreover, nanoparticle-loaded aliskiren decreased vasoconstriction of the mesenteric artery and collagen content (by 11%), and CSA (by 25%) in the aorta compared to the powdered aliskiren group. In conclusion, nanoparticle-loaded aliskiren represents a promising drug with antihypertensive and cardioprotective effects.

## 1. Introduction

Hypertension or high blood pressure generally leads to heart attacks, heart failure, kidney disease, stroke, and death and is still a major global health concern. Treatment of hypertension can take a multipronged approach including diet changes, exercise, and medication [1,2,3]. There are many classes of antihypertensive drugs, which lower blood pressure by different means. The most widely used drugs include thiazide diuretics, ACE inhibitors, angiotensin II receptor antagonists, calcium channel blockers, and beta blockers [4,5]. There has also been extensive progress in the development of novel therapeutics, which target the renin-angiotensin-aldosterone-system (RAAS) [6,7,8].

Aliskiren is the most recent antihypertensive agent and acts by inhibition of renin, the first step in RAAS. Aliskiren binds to the S3bp binding site of renin, which is essential for its activity. By binding to this pocket, aliskiren prevents the conversion of angiotensinogen to angiotensin I [9]. This effect subsequently decreases the formation of angiotensin II, followed by a decrease in vasoconstriction, aldosterone secretion and catecholamine release resulting in a decrease in blood pressure [9]. Moreover, aliskiren has been shown to exert cardio-protective, reno-protective and anti-atherosclerotic effects independent of its blood pressure lowering activity [10,11,12]. Aliskiren is available as 150 mg or 300 mg tablets which should be administered daily. However, a higher dose (300 mg) of aliskiren was required to achieve a blood pressure reduction comparable with that of losartan or ACE inhibitors. Thus, the limiting factor for the treatment might be the relatively low bioavailability of aliskiren (2–7%) [13,14]. Relatively high dose and frequency of the treatment, which is needed for beneficial effects of the drug, may incur several side effects such as high blood potassium levels, particularly when used with ACE inhibitors in diabetic patients, low blood pressure in volume-depleted patients, angioedema, and others [8,15]. We hypothesized that gradually released aliskiren from polymer-based nanoparticles could at least partially solve the problem of bioavailability and frequency of the treatment. Therefore, we used a lower dose of aliskiren (25 mg/kg/day) in our experiment than is usual in other studies [16,17].

In fact, different nanoparticulate systems like lipid-based and polymeric nanoparticles have been analyzed to overcome the limitations associated with the bioavailability and frequency of the treatment [18,19]. Polymer-based nanoparticles, which have been extensively studied for oral antihypertensive drugs, include polylactide acid (PLA), poly-e-caprolactone (PCL), polylactide-*co*-glycolide (PLGA), eudragit RL/RS, hydroxy propyl methyl cellulose (HPMC), and chitosan [20,21,22]. 

The aim of our study was to analyze the effect of PLA nanoparticle-loaded aliskiren on blood pressure, nitric oxide synthase activity, the vasoactivity of the mesenteric artery and structural alterations of the heart and aorta that developed due to spontaneous hypertension in rats.

## 2. Results

### 2.1. Polymeric Nanoparticles: Preparation and Characterization

**Figure 1:**
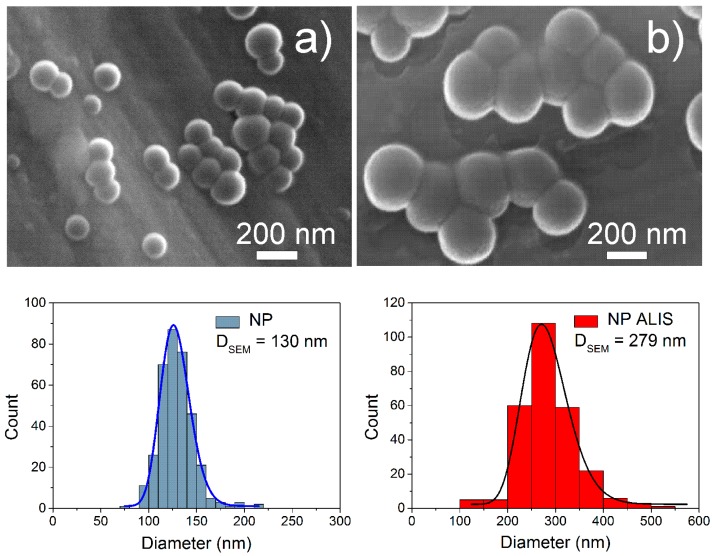
SEM images of nanoparticles (NP) (**a**) and nanoparticle-loaded aliskiren (NP ALIS) (**b**) with corresponding histograms of particle size obtained from the SEM images.

The most commonly used technique for the preparation of PLA nanoparticles (NP) is the modified nanoprecipitation method. Preparation of PLA nanoparticle-loaded aliskiren (NP ALIS) with the concentration of 5 mg ALIS/100 mg PLA was described in our previous study [23]. In this section of the article we have focused on detailed nanoparticle characterizations. Figure 1 shows the morphological characterization of NP and NP ALIS obtained by scanning electron microscope (SEM) with histograms showing particle size distributions. The studied nanoparticles in both samples were roughly spherical in shape. The particle size increase with ALIS encapsulation into NP was observed (Figure 1, Table 1); the value of D_SEM_—mean nanoparticle diameter determined from the lognormal fit of the histogram was raised from 130 nm (NP) to 279 nm (NP ALIS). The size distributions by intensity confirmed an increase in hydrodynamic size after aliskiren encapsulation from 147 nm (NP) to 253 nm (NP ALIS) as well. Furthermore, as can be seen in Table 1, all particle systems exhibit a narrow size distribution because their polydispersity index (PDI) is below 0.2. PDI is a measure of the broadness of the size distribution in the range from 0 to 1. The same increasing tendency of the diameter (D_DCS_) was observed in results obtained by differential centrifugal sedimentation (DCS) measurements. Table 1 summarizes the results from the sizing techniques that were used. The measured diameters are in the same nanoscale range. The discrepancies in obtained diameters are caused by the use of different sizing techniques with different physical principles, while SEM provides morphological information about nanoparticles and the consequent determination of size based on defined measures of diameter, dynamic light scattering (DLS) and DCS allow direct particle size analysis. The other reason could be in the state of the samples; SEM requires dried samples whereas DLS and DCS measure samples in a diluted liquid state. 

**Figure 2:**
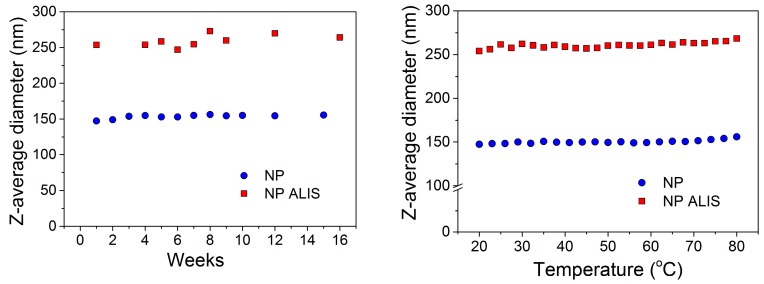
Time stability (**left**) and temperature stability (**right**) of NP and NP ALIS measured by DLS.

Furthermore, zeta potential measurements were carried out to obtain information about surface charge and stability of the prepared samples. In the absence of the drug, the zeta potential of naked/blank NP was about −17.0 mV. In the presence of drug, the negative surface charge was −24.6 mV, suggesting that part of the drug was incorporated within the polymer matrix and the rest was on the polymer surface. Time stability was confirmed by measuring the hydrodynamic size of all tested samples over a period of more than 16 weeks. The hydrodynamic size of nanoparticles remained at the original value with no evidence of flocculation or settling (Figure 2, left). In addition, the monitoring of temperature stability also showed that no thermal induced agglomeration has occurred in the tested samples. These results suggest that the preparation of the nanoparticle samples generates a stable complex that resists temperature increases (Figure 2, right).

**Figure 3:**
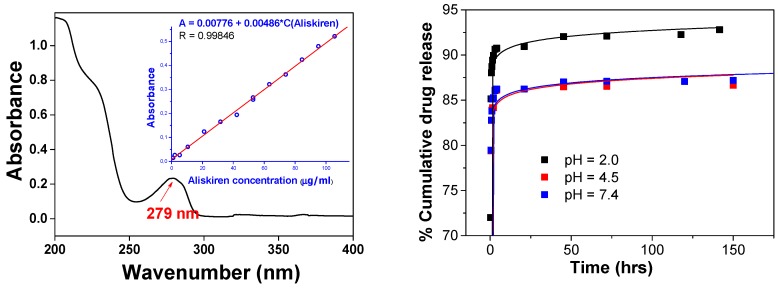
UV/vis spectrum of aliskiren (**left**) and calibration curve of pure aliskiren (inset). Time dependence drug release from NP ALIS at 37 °C and at different pH (**right**).

The amount of released drug was measured spectrophotometrically at *λ* = 279 nm (see Figure 3, left). As a result, time dependency of the released drug to the medium was obtained (Figure 3, right). The effect of pH (pH 2.0, 4.5, and 7.4) on the drug release profiles was also investigated. It was found that within 24 h, more than 85% of the total drug was released to the surrounding medium in the case of phosphate buffer pH 7.4, while more than 90% was released in the case of pH = 2.0. From the shape of the curve, it can be seen that the release took place in two phases. In the first steep phase, the drug adsorbed on the nanoparticles surface layers was released (up to about 75 min), while the second slower phase release is attributed to aliskiren entrapped in the PLA matrix, where the drug has to diffuse through the polymer layer. 

### 2.2. Blood Pressure and Relative Heart Weight

**Figure 4:**
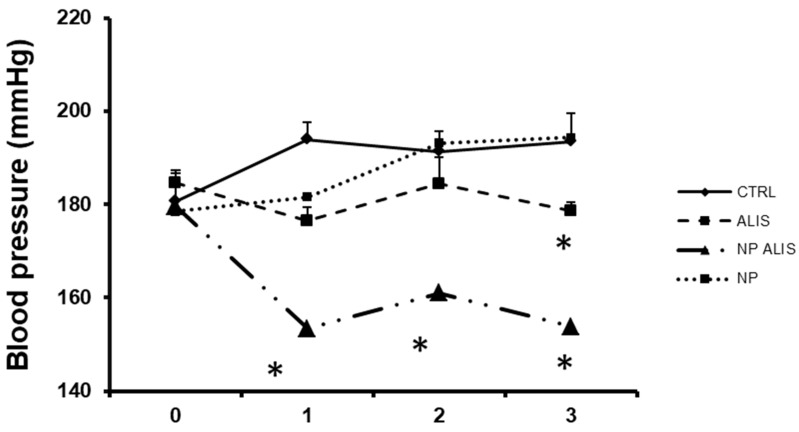
Effect of powdered aliskiren (ALIS), nanoparticle-loaded aliskiren (NP ALIS), and nanoparticles only (NP) on blood pressure of spontaneously hypertensive rats (CTRL), * *p* < 0.01 vs. CTRL; Values represent mean ± SEM of 6 animals.

The BP of the control SHR was 181 ± 7 mmHg at the beginning and reached 195 ± 8 mmHg at the end of the experiment. There were no significant changes in BP during nanoparticles only treatment. Powdered aliskiren decreased blood pressure by 10% and NP ALIS by 25% at the end of the treatment (Figure 4). Body weight, heart weight and HW/BW ratio are described in Table 2. There were no significant changes within the groups.

### 2.3. Vasoactivity of Mesenteric Artery

**Figure 5:**
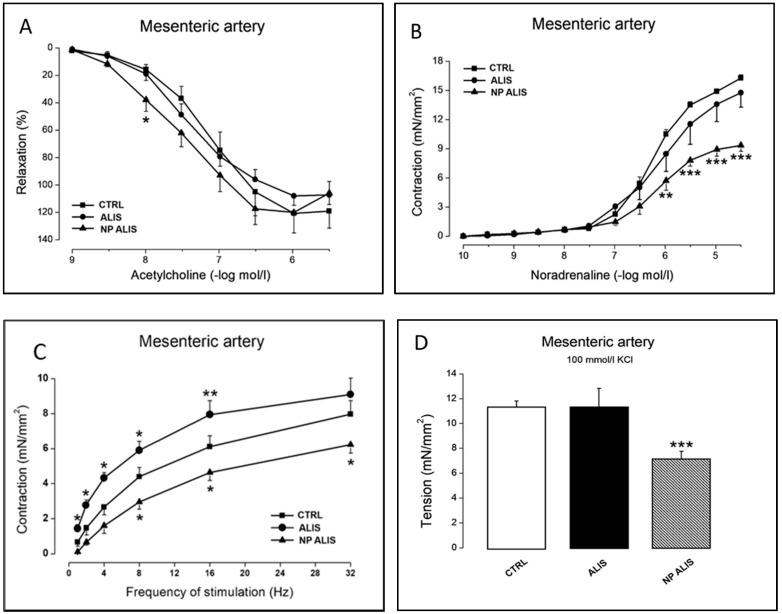
Effect of powdered aliskiren (ALIS) and nanoparticle-loaded aliskiren (NP ALIS) on vasoactivity of mesenteric artery. Endothelium-dependent relaxations (**A**), contractions induced by exogenous noradrenaline (**B**), neurogenic contractions (**C**), and contractions induced by high concentration of KCl (**D**). Values represent mean ± SEM of 6 measurements. Significant differences: * (**) (***) *p* < 0.05 (0.01) (0.001) ALIS or NP ALIS vs. CTRL.

Endothelium-dependent relaxations elicited by acetylcholine were moderately improved in mesenteric arterial preparations from SHR treated with NP ALIS when compared to untreated controls. This effect was not detected in mesenteric arteries from the powdered aliskiren group (Figure 5A). The values of acetylcholine concentrations required to produce half-maximal relaxant responses (EC_50_) did not differ between particular experimental groups (CTRL: 45 ± 12 nmol/L; ALIS: 32 ± 8 nmol/L; NP ALIS: 18 ± 7 nmol/L). The magnitude of phenylephrine-induced precontraction in arteries from NP ALIS-treated SHR was significantly smaller (3.44 ± 0.10 mN/mm^2^) when compared to control arterial preparations (4.93 ± 0.12 mN/mm^2^); however, in the powdered aliskiren group, the contractile response to 10^−6^ mol/L phenyleprine (4.86 ± 0.09 mN/mm^2^) did not differ from controls.

Contractile responses of mesenteric arteries are shown in Figure 5B–D. Administration of powdered aliskiren to SHR did not significantly affect the dose-response curves to noradrenaline and contractile responses to KCl in their mesenteric arteries. NP ALIS reduced the contractions of mesenteric arteries induced by exogenous noradrenaline and by a high concentration of KCl (Figure 5B,D). Similarly, neurogenic contractions of the mesenteric arteries induced by electrical stimulation of the perivascular nerves were reduced in SHR receiving NP ALIS, but not in comparison to arterial neurogenic contractions in rats treated with powdered aliskiren (Figure 5C). The values of EC_50_ for contractile responses to exogenous noradrenaline were not changed in mesenteric arteries from rats treated by powdered (701 ± 68 nmol/L) or nanoparticle-loaded aliskiren (598 ± 36 nmol/L) when compared to untreated rats (559 ± 18 nmol/L).

### 2.4. Total NOS Activity and NOS Isoforms Protein Expressions

**Figure 6:**
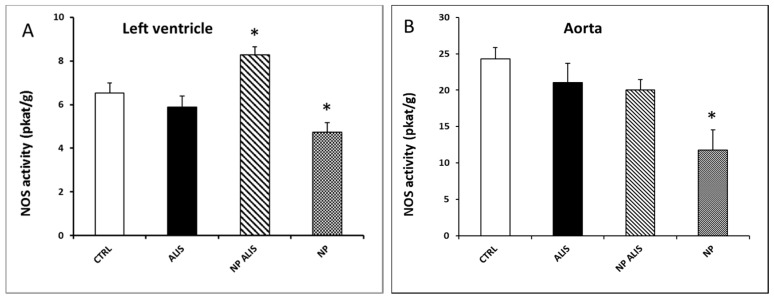
Effect of powdered aliskiren (ALIS), nanoparticle-loaded aliskiren (NP ALIS), and nanoparticles only (NP) on total nitric oxide synthase (NOS) activity in the left ventricle (**A**) and aorta (**B**). * *p* < 0.01 vs. CTRL; Values represent mean ± SEM of 6 animals.

Only NP ALIS significantly increased total NOS activity in the left ventricle (LV) in comparison to the control SHR. On the other hand, nanoparticles only decreased the level of total NOS activity in this tissue (Figure 6A). NOS activity in the aorta was not changed significantly within any aliskiren treated groups, however it was decreased in the nanoparticle only treated group (Figure 6B).

**Figure 7:**
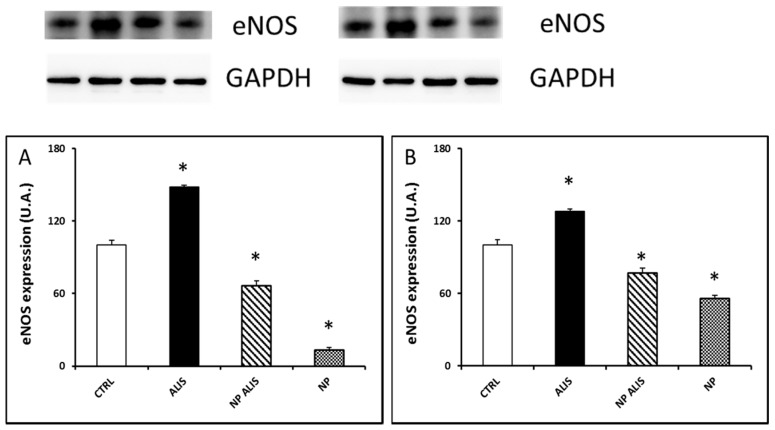
Effect of powdered aliskiren (ALIS), nanoparticle-loaded aliskiren (NP ALIS), and nanoparticles only (NP) on endothelial nitric oxide synthase (eNOS) protein expression in the left ventricle (**A**) and aorta (**B**). * *p* < 0.01 vs. CTRL; Values represent mean ± SEM of 6 animals.

**Figure 8:**
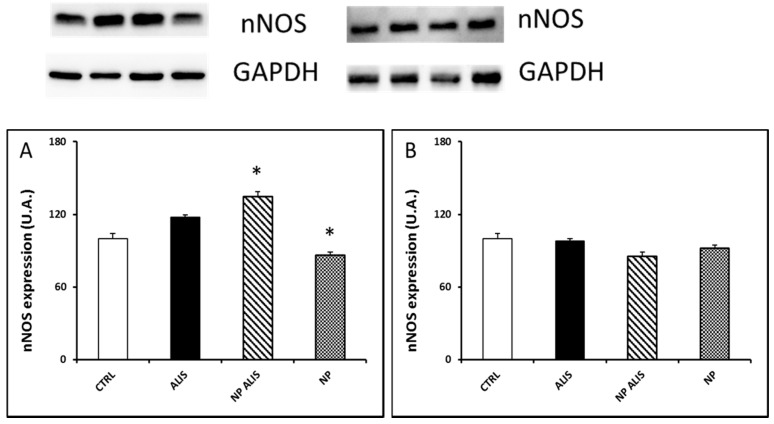
Effect of powdered aliskiren (ALIS), nanoparticle-loaded aliskiren (NP ALIS), and nanoparticles only (NP) on neuronal nitric oxide synthase (nNOS) protein expression in the left ventricle (**A**) and aorta (**B**). * *p* < 0.01 vs. CTRL; Values represent mean ± SEM of 6 animals.

Endothelial NOS protein expression was upregulated in the LV and aorta of the powder aliskiren group (by 48% and 28%, respectively). On the other hand, it was downregulated in the NP ALIS group (by 36% and 23%, respectively) and even more in the nanoparticles only group (by 87% and 44%, respectively, Figure 7A,B). Interestingly, nNOS protein expression in the LV was increased in the NP ALIS group by 34%. On the other hand, in the nanoparticle only group it was decreased by 24% (Figure 8A). There were no significant changes in nNOS protein expression in the aorta within the groups (Figure 8B).

### 2.5. Morphological Analysis

**Figure 9:**
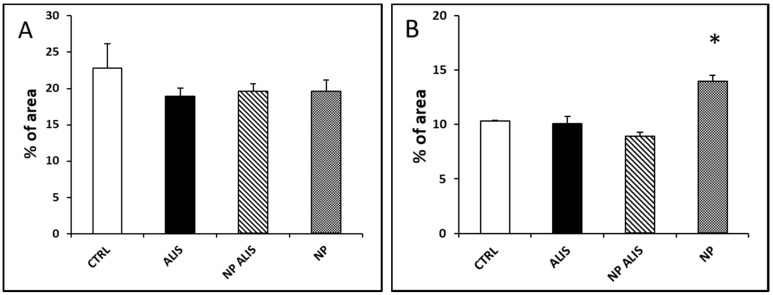
Effect of powdered aliskiren (ALIS), nanoparticle-loaded aliskiren (NP ALIS), and nanoparticles only (NP) on collagen content in the heart (**A**) and aorta (**B**). * *p* < 0.01 vs. CTRL; Values represent mean ± SEM of 6 animals.

Powdered aliskiren did not change collagen content in either the LV or in the aorta. NP ALIS significantly decreased collagen content only in the aorta. Surprisingly, treatment with nanoparticles only led to marked fibrosis of aortic tunica media (Figure 9A,B).

**Figure 10:**
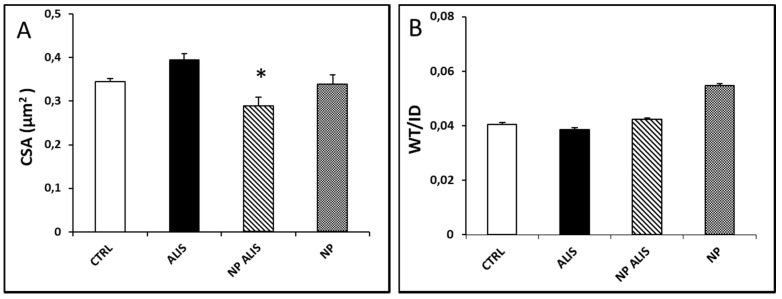
Effect of powdered aliskiren (ALIS), nanoparticle-loaded aliskiren (NP ALIS), and nanoparticles only (NP) on cross sectional area (CSA) (**A**) and wall thickness – inner diameter ratio (WT/ID) (**B**) of the aorta. * *p* < 0.01 vs. CTRL; Values represent mean ± SEM of 6 animals.

Neither powdered aliskiren nor NP ALIS changed elastin content in the aorta, however, nanoparticles only increased it significantly (data not shown). Cross-sectional area decreased significantly in the NP ALIS group, and wall thickness and WT/ID significantly increased in the nanoparticles only group (Figure 10A,B).

## 3. Discussion

In this study, we analyzed the suitability of polymer nanoparticles as drug delivery systems for aliskiren, and the effects of nanoparticle-loaded aliskiren on the cardiovascular system in hypertensive rats. 

PLA (FDA, US Food and Drug Administration approved biodegradable polymer) was selected as polymer matrix due to its very low toxicity. The NP and NP ALIS were synthesized by a modified nanoprecipitation method and characterized by different sizing techniques (SEM, DLS and DCS). After drug encapsulation, the average diameter values increased. We assume that the increase in size is related to successful aliskiren encapsulation into PLA nanoparticles. The obtained PDI was similar for both samples and the values indicate a narrow size distribution. Moreover, the change of zeta-potential value from −17.0 mV (NP) to −24.6 mV (NP ALIS) indicated improving NP ALIS stability in comparison with NP, and suggesting that a part of the drug was incorporated into the polymer. Monitoring of the time and temperature sample stability showed that both NP and NP ALIS samples were colloidal stable in aqueous solutions over several weeks after their preparation. Moreover, they were thermally stable up to 80 °C. 

The in vitro release profile of aliskiren from NP PLA exhibited a typical biphasic release process. The rapid initial aliskiren release was probably due to drug molecules adsorbed or close to the surface of the nanoparticles, whereas the slower release profile was a typical sustaining release and would mainly depend on the drug diffusion and the matrix erosion, which is a slower process [24,25]. The in vitro release also indicated that the release property of aliskiren from nanoparticles not only depended on adsorption of the drug but also on diffusion through the PLA matrix.

Furthermore, we analyzed the effects of nanoparticle-loaded aliskiren on the cardiovascular system in spontaneously hypertensive rats. Studies in animals and humans indicate that aliskiren blocks the intrarenal RAAS, and interferes with harmful cellular effects of angiotensin II. The mechanisms of aliskiren action include enzymatic blockade of renin and prorenin at the site of the (pro)renin receptor, finally leading to a decrease in blood pressure. In patients with diabetic nephropathy, adding aliskiren to losartan resulted in an additional 20% reduction in urinary protein excretion as well [26]. A total of 13 randomized controlled trials with 12,222 patients indicated that aliskiren in combination therapy with angiotensin-converting enzyme inhibitors or angiotensin II receptor blockers had remarkable effects in reducing both systolic and diastolic blood pressure when compared with angiotensin-converting enzyme inhibitors or angiotensin II receptor blockers monotherapy, but with significantly increased risk of hyperkalaemia and kidney injury. Relatively high dose (300 mg/kg/day) of aliskiren was used in the majority of trials [27]. In the animal study using SHR, aliskiren at high (60 mg/kg/day), but not low dose (30 mg/kg/day) prevented age-related increases in blood pressure [28]. In our experimental study, the blood pressure was measured by non-invasive tail-cuff plethysmography. In order to verify the correctness of the tail-cuff measurements in rats, we previously analyzed the relationship between directly recorded systolic pressure using carotid arterial cannulas and simultaneously recorded indirect tail-cuff blood pressure. We obtained r = 0.962 degree of correlation (unpublished results). Similarly, Pffefer et al. [29] measured the same relationship using 10 normotensive and 5 spontaneously hypertensive rats. The high degree of correlation obtained (r = 0.975) over a wide pressure range (62 to 263 mmHg) indicated that valid tail-cuff systolic pressures can be obtained in unanesthetized rats providing standard measurement conditions [29]. Our results demonstrated that nanoparticle-loaded aliskiren decreased blood pressure by 25%, while the powdered one decreased BP by only 10%. It seems that PLA nanoparticles may significantly decrease the dose along with increasing efficiency of aliskiren, and has the potency to decrease side effects of the drug. Three weeks of treatment did not, however, decrease the relative heart weight (see Table 2). 

Similarly, colon targeted methacrylic acid copolymeric nanoparticles ameliorated oral bioavailability of nisoldipine [30]. For delivering nifedipine, the authors prepared three different nanoparticles of PCL, PLGA, and eudragit. Significant reduction in blood pressure was seen with PCL NP (189 ± 2 mmHg to 12  ± 2 mmHg) and PLGA NP (113 mmHg ± 2 mmHg [30]. Shah et al. [31] tested PLGA nanoparticles with felodipine, which normalized systolic blood pressure and elevated ST segment of ECG under control compared to the drug suspension [31]. Oduk et al. (2018) [32] demonstrated that nanoparticle-mediated delivery even increases the angiogenic and therapeutic potency of vascular endothelial growth factor for the treatment of ischemic heart disease [32]. Recently it was documented that chitosan based antihypertensive nano-ceuticals can improve oral bioavailability and increase the plasma half-life of antihypertensive drugs by their sustained release in the lower part of the gastrointestinal tract [33]. Innovative NO-releasing polymeric nanomaterials also represent a big challenge in the development of qualitatively new antihypertensive drugs [34]. Interestingly, in our experimental study, NP ALIS was able to increase NOS activity in the left ventricle, which may contribute to blood pressure reduction in the NP ALIS group.

Similarly, endothelium-dependent relaxations elicited by acetylcholine were improved in mesenteric arteries from SHR treated with NP ALIS when compared to untreated controls. These results corresponded very well with a more pronounced blood pressure decrease after NP ALIS treatment compared to powdered aliskiren. Gu et al. [35] have shown improved endothelium-dependent relaxations in the thoracic aorta after powdered aliskiren administration at the concentration of 60 mg/kg/day, while lower concentration (30 mg/kg/day) was without effect. In our study, nanoparticle-loaded aliskiren was able to improve endothelium-dependent relaxations at a dose of 25 mg/kg/day, suggesting better drug efficacy. It must be noted, however, that due to technical problems, only two vasoreactivity measurements of the NP alone group were completed. Because of the inconsistent results obtained, we are not able to proclaim whether the nanoparticles alone might have an unfavorable effect on arterial reactivity, or not.

Activation of PI3K/Akt/eNOS signal pathway as one of the mechanisms leading to the improvement of vasorelaxation after aliskiren treatment has been assumed [35]. In our experiments, total NOS activity was, however, significantly increased only in the heart and upregulated nNOS protein expression probably contributed to this activity increase. Interestingly, increased eNOS protein expression in the heart and aorta after powdered aliskiren treatment did not lead to an increase in NOS activity. More interestingly, decreased eNOS protein expressions were seen in both NP ALIS and NP only groups. We hypothesize that the PLA nanoparticles that we used may damage endothelium and/or lead to NOS uncoupling. Indeed, results of studies described in the review of Cao et al. [36] indicated that nanoparticles could be internalized into endothelial cells by the endocytosis pathway as well as transported across endothelial cells by exocytosis and paracellular pathways. Interaction of endothelial cells with nanoparticles could induce genotoxicity, cytotoxicity, and eNOS uncoupling, which could be explained by nanoparticle-induced oxidative stress and inflammatory responses. In addition, some studies have also evaluated the influences of the microenvironment such as physiological and/or pathological stimuli related to the variety of endothelial cells, e.g., cyclic or shear stress and inflammatory stimuli [36]. This fact could explain decreased eNOS protein expression while nNOS protein expression increased or did not change after NP ALIS treatment. Despite the damaging effect of nanoparticles on endothelial cells and/or eNOS, NP ALIS was able to increase NOS activity in the heart. Thus, nanoparticles with better endothelium-related biocompatibility are needed to accelerate the beneficial effect of the drug. Mass, size, and surface interactions of nanoparticles with endothelium may affect the final effect of the drug. Similarly, silica nanoparticles showed a concentration- and size-dependent toxic effect that is endothelium specific and may affect the relaxation function of the vessels [37].

NP ALIS also significantly decreased collagen content in the aorta. Ferri et al. [38] demonstrated the inhibitory action of aliskiren on smooth muscle cell migration induced by prorenin. The inhibitory effect of aliskiren on fibroblast proliferation in AGT-REN double transgenic hypertensive mice in vitro has been documented as well [39]. These results correspond well with our analyses. Surprisingly, treatment with nanoparticles only led to marked fibrosis of aortic tunica media. We hypothesize that damaged endothelium due to nanoparticles incorporation may be partly responsible for this effect.

## 4. Materials and Methods 

### 4.1. Chemicals

Most of the chemicals and reagents were obtained from Sigma-Aldrich (Saint-Louis, MO, USA); when not, the company is indicated.

### 4.2. Polymeric Nanoparticles Preparation and Characterization 

The preparation and physicochemical characterizations of all tested samples were described in detail in our previous study [23]. Briefly, the modified nanoprecipitation method was used for aliskiren encapsulation into PLA nanoparticles (NP ALIS). After optimization study, NP ALIS theoretically loaded with 5% *w*/*w* aliskiren were prepared and used for the next in vivo experiments. The amount of non-encapsulated drug in the supernatant was determined by UV/VIS spectroscopy [23]. The samples were washed 3 times by ultracentrifugation in order to remove free aliskiren and finally, were dispersed into a known volume of pure water or of appropriate buffer solution and used for the experiments that are described later. Aliskiren free nanoparticles (NP) were prepared according to the same procedure, omitting the drug. To determine the exact mass of nanoparticles in 1 mL of suspension, a known volume of the nanoparticle sample was freeze–dried and weighed.

To obtain reliable data about the physicochemical properties of nanoparticles, it is advisable to use different techniques that work on different principles to provide us with complementary information. By using a scanning electron microscope (SEM, JEOL 7000F, Tokyo, Japan), the shape and particle size of the NPs were determined. A droplet of the water-diluted colloidal dispersion was deposited on a metal sample stub and dried under vacuum prior to sputtering with carbon and subsequent observation by SEM. For diameter determination, about 250 individual nanoparticles were analyzed; the resulting size distribution was fitted with a log-normal function. 

The next sizing technique used for the particle size distributions, time and thermal stability studies, was a dynamic light scattering (DLS). DLS measures the time-dependent fluctuations in scattered light from nanoparticles in a solution to determine the translational diffusion coefficient, and subsequently, the hydrodynamic size (D_DLS_) from the Stokes-Einstein equation. The DLS measurements were performed on the Zetasizer Nano ZS (Malvern Instruments) at 25 °C. The stability of the nanoparticle samples was evaluated by monitoring their hydrodynamic diameters as a function of time and temperature as well. Thermal stability measurements were performed between 20 °C and 80 °C. Moreover, zeta potential was measured by the same equipment. Zeta potential is commonly used to assess the stability of colloidal systems. 

Differential centrifugal sedimentation (DCS) was applied as a complementary method to DLS. DCS is an extremely powerful tool for high resolution particle characterization. The principle of the method is based on measuring particles sedimentation in a fluid. Stokes’ law is used to determine an unknown distribution of spherical particle sizes by measuring the time required for the particles to settle a known distance in a fluid of known viscosity and density. The samples were injected into the center of the rotating disc of a disc centrifuge (DC UHR 24000, CPS Instruments, Inc., Prairieville, LA, USA) at the beginning of the analysis. The time for particles to reach the detector beam versus beam intensity was converted to a size distribution using both Stokes’ law (modified slightly for use in a centrifuge) and Mie theory light scattering calculations.

The drug release was investigated by the external sink method based on the utilization of a release medium which is a good solvent of the studied drug and an absolute non-solvent of the particle-forming polymer [40]. A part of the prepared nanoparticles was freeze-dried, dispersed in drug release solutions (phosphate buffer) and consequently shaken and incubated, maintaining a temperature of 37 °C. Then, the dispersion was centrifuged, and fixed quantities of supernatant (containing released drug) was removed and replaced with the same quantity of fresh medium. The amount of released drug was measured spectrophotometrically at wavelength 279 nm.

### 4.3. Animals and Treatment

All procedures and experimental protocols were performed in accordance with institutional guidelines and were approved by the State Veterinary and Food Administration of the Slovak Republic (Ro-1998/15-221) and by an Ethical committee of the Institute of Normal and Pathological Physiology Slovak Academy of Sciences according to the European Convention for the Protection of Vertebrate Animals used for Experimental and other Scientific Purposes, Directive 2010/63/EU of the European Parliament. All rats used in the study were born in an accredited breeding establishment. They were housed in groups of 3 animals, under a 12 h light–12 h dark cycle, at a constant humidity (45–65%) and temperature (20–22 °C), with free access to standard laboratory rat chow and drinking water. 

Twelve week-old male spontaneously hypertensive rats (SHR) were divided into an untreated group, a group treated with powdered (25 mg/kg/day) or nanoparticle-loaded aliskiren (25 mg/kg/day), and a group treated with nanoparticles only. Each group consisted of 6 animals. Treatment was administered via gavage for 3 weeks. Daily water consumption was estimated individually for every animal and adjusted, if necessary. All animals were housed at a temperature of 22–24 °C and fed with a regular pellet diet ad libitum. Blood pressure was measured non-invasively, using tail-cuff-plethysmography weekly. At the end of treatment, the animals were sacrificed, body weight (BW) and heart weight (HW) were determined. Relative heart weight was calculated as a HW/BW ratio. Samples of the left ventricle and aorta were used to determine NOS activity, endothelial NOS and neuronal NOS protein expressions by Western blot analysis. Samples of the heart and aorta were taken for morphological analysis and mesenteric artery for vasoactivity measurement.

### 4.4. Measurement of Vasoactivity

The superior mesenteric arteries were dissected out from rats and carefully removed of adhering connective tissue. From each artery, one ring segment of approximately 3 mm length was cut out, with special care to preserve intact endothelium. Then, arterial rings were suspended in 20 mL organ baths filled with oxygenated (95% O_2_ + 5% CO_2_) modified Krebs solution maintained at 37 °C and set up for isometric tension recording using a force-displacement transducer Sanborn FT 10 (Sanborn, Baltimore, MD, USA). The Krebs solution was prepared in the following composition (in mmol/L): NaCl 118, KCl 5, CaCl_2_ 2.5, MgSO_4_ 1.2, NaHCO_3_ 25, KH_2_PO_4_ 1.2, glucose 11, CaNa_2_.EDTA 0.03. The preparations were equilibrated under a resting tension of 10 mN for 60–90 min, and the Krebs solution was changed every 15 min.

Acetylcholine-induced relaxations in mesenteric arteries were measured on phenylephrine-precontracted preparations: when a plateau tension had been achieved after submaximal concentration with phenylephrine (10^−6^ mol/L), the vessels were relaxed by exposure to stepwise increases in acetylcholine concentration (10^−9^ mol/L–3 × 10^−6^ mol/L).

Adrenergic contractions were determined in mesenteric arterial preparations as the responses to cumulatively applied exogenous noradrenaline (10^−10^mol/L–3 × 10^−5^ mol/L) or as the neurogenic responses elicited by electrical stimulation of periarterial sympathetic nerves. The arterial rings were stimulated by two parallel platinum plate electrodes placed on either side of the preparation and connected to an electrostimulator ST-3 (Medicor, Budapest, Hungary). Frequency-response curves to electrical stimuli were obtained using square pulses of 0.5 ms in duration, at a supramaximal voltage (>30 V), applied at 1–32 Hz, for a period of 20 s. In mesenteric arteries, contractions to 100 mmol/L KCl were also determined.

Relaxations to acetylcholine were expressed as relative values (in % of phenylephrine precontraction). Contractile responses were expressed as absolute values; the levels of force (in mN) were normalized to the cross-sectional areas (in mm^2^), which were calculated from wet weight and circumference of the respective arterial preparations using the following equation: cross-sectional area (mm^2^) = [wet weight (mg)]/[1.06 mg/mm^3^ × circumference (mm)]; where 1.06 mg/mm^3^ represents the estimated density of vascular tissue.

### 4.5. Total NOS Activity and NOS Isoforms Protein Expressions

Total NOS activity was determined in crude homogenates of the left ventricle and aorta by measuring the formation of [3H]-l-citrulline from [3H]-l-arginine (ARC, Montana, USA) as previously described and slightly modified by Pechanova et al. [41]. [3H]-l-citrulline was measured with the Quanta Smart TriCarb Liquid Scintillation Analyzer (Packard Instrument Company, Meriden, CT, USA). 

Protein expressions of eNOS and nNOS were determined in the left ventricle and aorta by Western blot analysis. The samples were probed with polyclonal rabbit, anti-eNOS and anti- nNOS and anti-GAPDH antibodies (Abcam, Cambridge, UK). The intensity of bands was visualized using the enhanced chemiluminescence system (ECL, Amersham, UK), quantified by using ChemiDocTM Touch Imagine System (Image LabTM Touch software, BioRad, Hercules, CA, USA) and normalized to GAPDH bands. 

### 4.6. Morphological Analysis

Samples of the left ventricle and aorta were fixed in 10% buffered formalin solution at room temperature and processed by the standard paraffin technique for light microscopy examination. Aortas were processed embedded in one agarose block array. Five um thick tissue sections were stained with hematoxylin–eosin (H-E), Weigert elastin stain (Weigert) and picrosirius red (PSR), thereafter examined and scored. Histopathological evaluation was performed in four sections per slide for all specimens. All morphological observations were performed on a NIKON Eclipse Ti C2+ microscope (Tokyo, Japan).

### 4.7. Morphometric Analysis of the Aorta

H-E stained aorta array sections were scanned by 10× objective in transmitted light as one large image. Thereafter, we manually labeled tunica media borders of the aorta in NIKON NIS-Elements Analysis software. After the labeling, the inner and outer diameter and wall thickness were automatically measured and calculated by software. CSA was calculated as the area bounded by the inner and outer tunica media diameter.

### 4.8. Collagen and Elastin Content

PSR stained tissue slices were observed by polarized light microscopy. The whole area of every tissue section was scanned by automatic scanning utility on Eclipse Ti with 20× objective. After exclusion of damaged parts of the scan, 16 fields were selected randomly for every section. Color image analysis was performed by ImageJ morphometric software and fibrosis enlargements were expressed as the area of positive signal toward the whole field of view area in polarized light.

### 4.9. Statistics

The results are expressed as mean ± S.E.M. One-way, two-tailed analysis of variance and Duncan test were used for statistical analysis. Values were considered significant with a probability value *p* < 0.05 (for both ANOVA and Duncan test). *p* values were multiplicity adjusted.

## 5. Conclusions

The efficacy and safety of aliskiren in comparison to other antihypertensive drugs have been studied in many clinical trials and meta-analyses. Several large trials have attempted to show the potential benefits of aliskiren beyond its blood pressure lowering activity, as well as morbidity and mortality outcomes in patients with heart failure, diabetes mellitus, and post-myocardial infarction patients. However, some recent trials have demonstrated several side effects of aliskiren, especially when used in combination with angiotensin-converting enzyme inhibitors in patients with diabetes mellitus. Relatively high doses of aliskiren used for the treatment may partially contribute to the drug’s side effects. Our results indicated that PLA nanoparticles may significantly decrease the dose along with increasing efficiency of aliskiren with the potency to decrease the side effects of the drug as well. Our study also indicated that nanoparticles with better endothelium-related biocompatibility are needed to accelerate the beneficial effect of aliskiren. Mass, size, and surface interactions are important candidates to be tested when interacting with endothelium. 

## Figures and Tables

**Table 1:** Characterization of NP, and NP ALIS. D_SEM_—mean nanoparticle diameter determined from SEM images; D_DCS_—mean nanoparticle diameter measured by differential centrifugal sedimentation (DCS); D_DLS_—mean nanoparticle diameter measured by DLS; PDI—polydispersity index.

Sample	Solid Concentration (mg/mL)	D_SEM_ (nm)	D_DCS_ (nm)	D_DLS_ (nm)	PDI	Zeta Potential (mV)
NP	92.3	130.2 ± 0.4	167	147	0.089	−17.0
NP ALIS	104	278.6 ± 1.2	300	253	0.087	−24.6

**Table 2:** Effect of powdered aliskiren (ALIS), nanoparticle-loaded aliskiren (NP ALIS), and nanoparticles only (NP) on body weight, heart weight and HW/BW ratio. Values represent mean ± SEM of 6 animals.

	BW (g)	HW (g)	HW/BW (× 10^−3^)
CTRL	296 ± 8	1.15 ± 0.04	3.88 ± 0.09
ALIS	303 ± 9	1.11 ± 0.02	3.67 ± 0.06
NP ALIS	284 ± 5	1.04 ± 0.05	3.66 ± 0.08
NP	279 ± 7	1.05 ± 0.03	3.76 ± 0.05
